# Hepatitis A virus infection associated with bilateral pleural effusion, ascites, and acalculous cholecystitis in childhood: a case report

**DOI:** 10.1186/s13256-024-04627-8

**Published:** 2024-06-26

**Authors:** Fatima Breim, Bakri Roumi Jamal, Lama Kanaa, Saleh Bourghol, Besher Jazmati, Samia Dadah

**Affiliations:** 1 Faculty of Medicine, University of Aleppo, Aleppo, Syria; 2 Department of Pediatric, Faculty of Medicine, University of Aleppo, Aleppo, Syria

**Keywords:** Pleural effusion, Ascites, Acalculous cholecystitis, Hepatitis A virus, Pediatric, Case Report

## Abstract

**Background:**

Acute hepatitis A infection is common among children in developing nations. The clinical presentation in children is usually asymptomatic and anicteric, and it is a self-limiting infection. Rarely, it can be associated with extrahepatic complications such as pleural effusion, acalculous cholecystitis, and ascites.

**Case presentation:**

An 8-year-old middle eastern child presented with abdominal pain, jaundice in the sclera, yellowish color of urine, and poor appetite. In the last two days, abdominal distension developed. After conducting diagnostic investigations, the child was diagnosed with HAV hepatitis associated with bilateral pleural effusion, acalculous cholecystitis, and ascites. He was managed conservatively with vitamin K supplementation and supportive parenteral fluids. After 4 days, clinical improvement was observed.

**Conclusion:**

Hepatitis A infections presented with extrahepatic manifestations like pleural effusion, acalculous cholecystitis, and ascites are very rare, especially in children. There have been some reports of these manifestations occurring in isolation, but for them to co-exist to our knowledge, this has only been reported in two cases in the literature, and this is the third case with all these three rare complications being presented simultaneously in a single child. Although HAV infection is an asymptomatic and self-limiting viral disease in childhood, it can manifest with rare extrahepatic complications, so pediatricians should be aware of this rare association to avoid unnecessary investigations.

## Introduction

Hepatitis A virus (HAV) causes liver inflammation, resulting in a range of symptoms from mild to severe, including fever, severe fatigue, loss of appetite, abdominal discomfort, nausea and vomiting. However, it is usually a self-limiting disease, especially in childhood [1, 2]. HAV is transmitted through physical contact and the fecal–oral route when contaminated food or water containing the feces of an infected person is ingested [2]. It is a widespread illness, especially in developing countries due to poor hygiene and sanitation practices [3]. It is infrequently accompanied by extrahepatic manifestations in children, such as acalculous cholecystitis, pancreatitis, pleural effusion, ascites, urticarial and maculopapular rash, and acute kidney injury [4].

Pleural effusion is a rare complication of hepatitis A that typically occurs in children during the early stages of the disease [3, 4]. Ascites is also a rare extrahepatic manifestation, tends to be commonly observed in the later stages of the illness [5].

We presented a case of an 8-year-old boy with hepatitis A virus infection who exhibited all three manifestations of ascites, acalculous cholecystitis, and pleural effusion simultaneously.

To the best of our knowledge, this is only the third reported case in the literature where all three complications have presented concurrently. The case highlights the importance of recognizing these manifestations in primary care settings to avoid unnecessary procedures.

## Case presentation

An 8-year-old middle eastern boy presented to the hospital with abdominal pain for the past 5 days and abdominal distention for the last 2 days. The patient remained hemodynamically stable with scleral icterus, yellowish urine, poor appetite, and vital signs showing a heart rate of 116 beats per minute, respiratory rate of 24 breaths per minute, and blood pressure of 120/80 mmHg. There was no fever, diarrhea, constipation, or significant medical or surgical history noted.

On physical examination, dullness in percussion and decreased breath sounds were noted on chest examination. The liver was found to be soft and palpable 3 cm below the costal margin, and the spleen was not palpable. Laboratory studies revealed a hemoglobin level of 9.8 g/dL, hematocrit of 29.0%, white blood cell count of 9.3 × 10^9^ cells/L, lymphocyte count of 5.1 × 10^9^ cells/L, and platelet count of 221 × 10^9^ cells/L. The total protein level was 6.60 g/dL, with an albumin level of 3.13 g/dL, and prothrombin time was 17.20 s. The patient also had hyperbilirubinemia with a total bilirubin level of 6.54 mg/dL and direct bilirubin level of 6.35 mg/dL, transaminitis with aspartate aminotransferase of 741.5 U/L, and alanine aminotransferase of 805.7 U/L (Table 1).

**Table 1:** Patient’s laboratory test result on admission

Laboratory test	Reference range	On admission
Hgb (g/dl)	11.5–16.5	9.8
HCT %	35.0–55.0	29.0
WBC (10^9^ cells/L)	3.5–10.0	9.3
LYM (10^9^ cells/L)	0.5–5.0	5.1
PLT (10^9^ cells/L)	100–400	221
PT (Seconds)	12.5–15.0	17.2
Total proteins (g/dL)	6.6–8.7	6.60
Albumin (g/dL)	3.5–5	3.13
Total bilirubin (mg/dL)	Up to 5.0	6.54
Direct bilirubin (mg/dL)	0.0–0.25	6.35
AST (U/L)	10–40	741.5
ALT (U/L)	Up to 40	805.7
Anti-HAV Ab (IgM)	Up to 0.9 Borderline: 0.9–1.1 Positive: more than 1.1	7.63
Serologies for hepatitis B and C		Negative

*Hbg* hemoglobin level, *HCT* hematocrit, *WBC* white blood cell count, *LYM* lymphocytes, *PLT* platelet count, *PT* prothrombin time, *AST* aspartate aminotransferase, *ALT* alanine aminotransferase, *IgM* Immunoglobulin M

The IgM anti-HAV test was positive. Serologic tests for hepatitis B virus, hepatitis C virus, and hepatitis E virus were negative. Serological analysis for cytomegalovirus, parvovirus, leptospira, and Epstein Barr virus were also found to be negative. As a result, the diagnosis of HAV infection was confirmed.

**Fig. 1:**
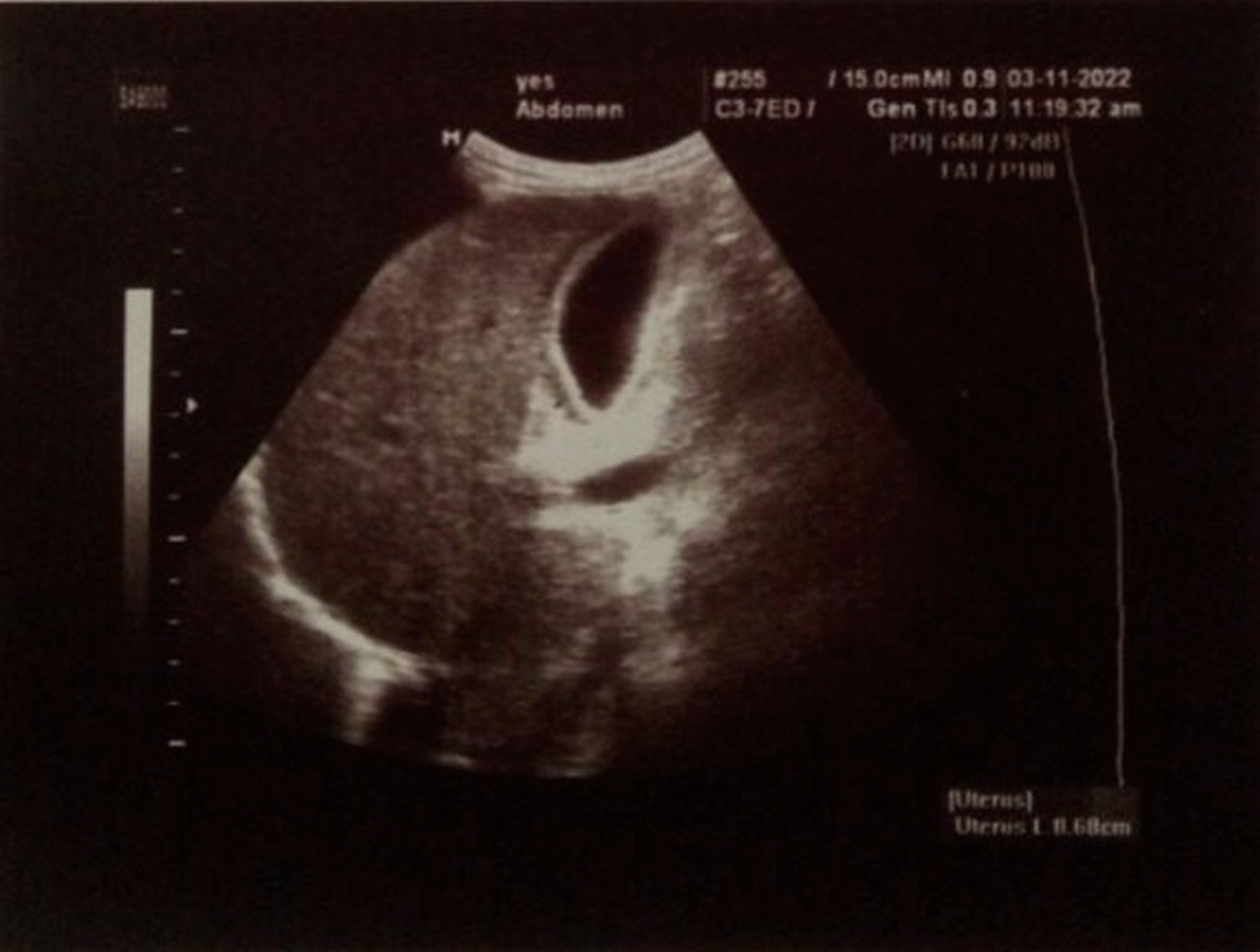
Thickening of the gallbladder wall and edematous pericholecystic area in abdominal ultrasonography

A chest X-ray revealed bilateral pleural effusion, with the left side exhibiting a greater effusion volume than the right side. Thoracic ultrasonography showed mild bilateral pleural effusion. Abdominal ultrasound indicated mild hepatomegaly and a thickened (7 mm), non-calculous gallbladder, suggestive of acalculous cholecystitis, as well as moderate free fluid in the abdomen (Fig. 1).

The patient was diagnosed with HAV acute hepatitis associated with bilateral pleural effusion and ascites, confirmed by X-ray imaging and ultrasonography. The patient received conservative management, including vitamin K supplementation and supportive parenteral fluids. Specifically, the patient received 5 mg of vitamin K twice daily for 3 days and was given 70% of the maintenance requirement of potassium chloride dissolved in a solution of 5% dextrose and sodium chloride intravenously for 2 days. He remained hemodynamically stable throughout the duration of illness.

After 4 days, a repeat ultrasound showed resolution of pleural effusion, ascites, and acalculous cholecystitis. Subsequent liver function tests and plain film chest X-ray revealed complete recovery over the course of a month. The patient did not experience any further complications or require additional treatment, and there were no long-term sequelae reported in this case.

## Discussion

HAV infection-induced acute hepatitis typically follows a benign, self-limiting course. The clinical manifestations and severity vary depending on age and are mainly associated with liver inflammation. In children, it is usually asymptomatic and not accompanied by jaundice; 80% of them recover completely in 3 months [4, 5].

Acute hepatitis caused by HAV infection may also present with rare extrahepatic manifestations, such as urticarial and maculopapular rash, acute kidney injury, autoimmune hemolytic anemia, aplastic anemia, acute pancreatitis, reactive arthritis, Guillain–Barre syndrome, pleural or pericardial effusion, ascites, glomerulonephritis, polyarteritis nodosa, thrombocytopenia, and cryoglobulinemia [4]. In childhood, pleural effusion, ascites, and acalculous cholecystitis are rare manifestations due to hepatitis A virus infection [3, 4].

Pleural effusion with hepatitis A virus infection was described in 20 case reports; most of them were children [4]. It has commonly been reported on the right side in patients during the early period of the disease. All cases were resolved spontaneously except one, which progressed to fulminant liver failure and led to death [4]. The exact mechanism of the effusion in hepatitis A virus infection is unknown; it appears to be multifactorial, potentially involving immune complex deposition or a direct viral invasion of the pleura. Additionally, there may be a secondary mechanism related to the transport of fluid from co-presenting ascites to the pleura cavity through small diaphragmatic defects or diaphragmatic lymphatics [3, 4, 6].

The association between hepatitis A and ascites has rarely been reported in both children and adults, with most cases documented in children at advanced disease stages. Possible mechanisms include venous and lymphatic obstruction due to liver involvement or a decrease in oncotic pressure caused by hypoalbuminemia [5].

Acalculous cholecystitis is a rare complication of acute viral hepatitis A. In children, it is usually asymptomatic and recovers within 3 weeks. The most common finding is gallbladder wall thickening greater than 3.5 mm. Suggested mechanisms include the direct effect of viral antigens [2, 3].

In our case, we reported hepatitis A complicated by pleural effusion, ascites, and acalculous cholecystitis. Pleural effusion due to pneumonia was considered less likely because the child had no history of coughing or tachypnea. Tuberculosis was ruled out due to the short duration of the illness and the absence of a contact history of tuberculosis. Surgical intervention was not required for acalculous cholecystitis because it was transient and gradually disappeared.

All three of these complications spontaneously resolved with supportive treatment in our patient.

To our knowledge, this is the third case with the presence of pleural effusion, ascites, and acalculous cholecystitis, three rare manifestations due to hepatitis A virus infection in a single child (Table 2) [1, 3].

**Table 2:** The previous two cases and our case who had all these three complications together

Characteristic	The first case [3]	The second case [1]	Our case
*Demographics*			
Sex	Male	Female	Male
Age (years)	12	3	8
Country	Turkey	India	Syria
*Symptoms*			
Ascites	Yes	Yes	Yes
Pleural effusion	Yes	Yes	Yes
Acalculous cholecystitis	Yes	Yes	Yes
Final diagnosis	HAV infection	HAV infection	HAV infection
*Lab tests*			
Hgb (g/dl)	10.9	11.6	9.8
WBC (10^9^ cells/L)	4.2	12.4	9.3
PLT (10^9^ cells/L)	172	216	221
PT (Seconds)	16	–	17.2
Total bilirubin(mg/dL)	6.3	3.5	6.54
Direct bilirubin (mg/dL)	5.6	1.5	6.35
AST (U/L)	1364	680	741.5
ALT (U/L)	1838	745	805.7
IgM anti-HAV	+	+	+
Serologies for hepatitis B and C	–	–	–
*Therapy*	Supportive parenteral fluids, a protein-lipid restricted, carbohydrate enriched diet, and one dosage of vitamin K	Supportive parenteral fluids, and one dosage of vitamin K	Supportive parenteral fluids, and three dosages of vitamin K

*Hbg* hemoglobin level, *WBC* white blood cell count, *PLT* platelet count, *PT* prothrombin time, *AST* aspartate aminotransferase, *ALT* alanine aminotransferase, *IgM* Immunoglobulin M

This case highlights the significance of recognizing extrahepatic manifestations of childhood hepatitis A in primary care settings. It underscores the importance of primary care physicians being knowledgeable about these manifestations to prevent unnecessary procedures like pleural and ascitic taps. When encountering cases with similar presentations, it is crucial to consider hepatitis A in the differential diagnosis. Management of such cases typically involves conservative approaches, including regular clinical assessments and monitoring through biochemical and radiological studies.

This work has been reported in line with the CARE criteria [7].

## Conclusion

The most important educational point of this study is that although HAV infection is an asymptomatic and self-limiting viral disease in childhood, it can manifest with rare extrahepatic complications. Additionally, hepatitis A infection should be considered in the differential diagnosis of pleural effusion, ascites, and acalculous cholecystitis in a patient with features of acute hepatitis, so pediatricians should be aware of this rare association to avoid unnecessary investigations.

## Data Availability

Not applicable.
